# Particle Size of Latex Beads Dictates IL-1β Production Mechanism

**DOI:** 10.1371/journal.pone.0068499

**Published:** 2013-07-09

**Authors:** Takumi Adachi, Kazuhiko Takahara, Jun Taneo, Yasuo Uchiyama, Kayo Inaba

**Affiliations:** 1 Department of Animal Development and Physiology, Graduate School of Biostudies, Kyoto University, Yoshida-Konoe, Sakyo, Kyoto, Kyoto, Japan,; 2 Japan Science and Technology Agency, Core Research for Evolutional Science and Technology (CREST), Tokyo, Japan,; 3 Department of Cell Biology and Neuroscience, Juntendo University Graduate School of Medicine, Bunkyo, Tokyo, Japan; Osaka University, Japan

## Abstract

Macrophages (Mϕ) are well documented to produce IL-1β through various signaling pathways in response to small particles such as silica, asbestos and urea crystals, in the presence of lipopolysaccharide (LPS). However, it has not been clear to what extent particle size affects the response. To investigate this point, we stimulated bone marrow-derived macrophages (BMDM) with size-defined latex beads (LxB). Although both nano-sized (20 nm) and micro-sized (1,000 nm) LxB induced IL-1β production, only the nano-sized particles formed large intracellular vacuoles. In contrast, 100 nm LxB did not induce either of the responses. The same cellular responses were also observed in primary microglia cells. Although K^+^ efflux and NLRP3 activation in BMDM were crucial in response to both 20 and 1,000 nm LxB, only IL-1β production by 20 nm LxB was sensitive to cathepsin B and P2X_7_, a receptor for ATP. The response by 1,000 nm LxB relied on a robust production of reactive oxygen species (ROS), since IL-1β production was remarkably reduced by ROS inhibitors such as diphenylene iodonium (DPI) and N-acetylcysteine (NAC). In contrast, IL-1β production by 20 nm LxB was augmented by NAC and in BMDM deficient in thioredoxin-binding protein-2 (TBP-2), a negative regulator of the ROS scavenger thioredoxin. These results suggest that the cells responded differently in their secretion of IL-1β depending on particle size, and that there is a range within which neither pathway works.

## Introduction

Cellular responsiveness to small (nano) particles is a critical issue in nanotechnology and cell biology (ref [1].) because of the ‘nano-effects’ such as cellular toxicity and tumorigenicity. The particles activate innate immune cells such as macrophages (Mϕ) and dendritic cells (DCs) *via* nucleotide binding oligomerization domain-like receptors (NLRs) [2], leading to sterile inflammation. In the NLRs, NOD-like receptor family, pyrin domain containing 3 (NLRP3), a component of NLRP3 inflammasome [3], has been revealed to be a potent sensor activated by particles such as silica [4], asbestos [5] and needle-like carbon nanotubes (CNT) [6] as well as endogenous monosodium urate crystals [7], cholesterin crystals [8,9] and amyloid complex [10,11].

Activation of the NLRP3 inflammasome by the particles results in the cleavage of the pro-form of caspase-1, an IL-1β converting enzyme, and leads to the robust production of active IL-1β, which is an endogenous pyrogen and a key cytokine for the early phase of inflammation. It has been observed that K^+^ efflux, reactive oxygen species (ROS) production, and leakage of cathepsins from destabilized phagolysosomes take place during the response to various test particles [4,12–17]. The cathepsin B released from phagolysosomes incorporating indigestible particles has been demonstrated to be a specific activator for NLRP3. Even though cathepsin B inhibitor suppresses IL-1β production by macrophages in response to silica [4,9,18], it has also been reported that cathepsin B-deficient Mϕ still respond to silica [9,19].

The particles have been also demonstrated to give rise to mitochondrial damage and a subsequent ROS production [13,16], leading to the release of thioredoxin-binding protein (TBP-2), a NLRP3 activator, from thioredoxin [15]. In these studies, the involvement of ROS in the IL-1β production was suggested as a result of using a ROS inhibitor, diphenylene iodonium (DPI), which has been widely used for the inhibition of ROS production. However, recent data suggest that DPI acts as an inhibitor for NLRP3 transcription [20] and NF-κB activation/signaling [21], possibly down-regulating the IL-1β production *via* ROS-independent pathways. Therefore, it is unclear whether the cathepsin-dependent and ROS-dependent pathways mutually cooperate or whether one or the other is exclusively utilized to produce IL-1β through NLRP3 activation.

Interestingly, Dostert et al. have reported that particulate heme-crystal (sub-micrometer ~ micrometer in size), hemozoin, induces IL-1β production *via* a ROS-dependent but cathepsin B-independent pathway [19]. In addition, Bruchard et al. have demonstrated that the anti-cancer drugs gemstabine and 5-fluorouracil, which lead to release of endosomal cathepsin B to cytosol and then the association of cathepsin B with NLRP3, evoke IL-1β production in a ROS-independent manner [22]. These results imply that ROS and cathepsin B impact NLRP3 activation independently under certain milieu.

Based on these observations, we assumed that the size of the test particles influenced cellular responsiveness to produce IL-1β. To address this question, we stimulated bone marrow-derived macrophages (BMDM) with various sizes of latex beads (LxB) in the presence of lipopolysaccharide (LPS), which induces pro-IL-1β through NF-B activation, and then measured the IL-1β production. Our study revealed that BMDM employed either of the pathways exclusively in response to whether the test particles were micro size or nanosize. Interestingly, we also found that there was a range of particle sizes within which no response was induced. These results suggest that cellular responses differ according to particle size and thus contribute to our understanding of the ‘nano-effects’ at the cellular level.

## Materials and Methods

### Ethics Statement

The animal experiments were carried out in strict accordance with the protocols approved by the Animal Experiment Committee of Graduate School of Biostudies, Kyoto University (Animal Experiment Protocol No. Lif-K12011). All efforts were made to minimize animal suffering.

### Mice and bone marrow cells

BALB/c and C57BL/6 mice were purchased from Japan SLC (Hamamatsu, Shizuoka, Japan). LC3-GFP knock-in mice [23], Parkin^-/-^ [24], TBP2^-/-^ [25] and NLRP3^-/-^ [26] mice were provided by Drs. N. Mizushima (Tokyo Medical and Dental University, Tokyo, Japan), R. Takahashi (Kyoto University, Kyoto, Japan), J. Yodoi (Kyoto University) and V. M. Dixit (Genentech, Inc., South San Francisco, CA), respectively. P2X_7_^-/-^ mouse bone marrows were from Dr. T. Ishibashi (Kyushu University, Fukuoka, Japan). Bone marrow of cathepsin B^-/-^ (CtsB KO) [27] and cathepsin D^-/-^ (CtsD KO) [28] mice was also used. All gene-modified mice were C57BL/6 background. Mice were kept under specific pathogen-free conditions and used at 8-12 weeks of age.

### Cell preparation and culture

BMDM were generated from bone marrow precursor cells in RPMI1640 containing 10% fetal calf serum, 50 µM 2-mercaptoethanol (culture medium) and 15% L929 cell culture supernatant for 5-6 days and stored at -80°C until use. After thawing the frozen cells, BMDM were cultured for 2 days in the same medium followed by detaching with 5 mM EDTA and were then subjected to the experiments. Primary microglia cells were prepared from postnatal mouse brains as described [29]. More than 95% of the cells were CD11b^+^ and CD45^int-low^.

For cytokine production, cells were plated at 3 x 10^4^ cells/100 µl/well of 96-well flat-bottomed plates or placed in 1.5 ml microtubes for stirred culture with rotation at 1 rpm (9.5 cm radius) using rotator RT-5 (TAITEC Co., Ltd., Saitama, Japan). Cells were stimulated with fluoresceinated or non-fluoresceinated carboxyl-LxB from Invitrogen (Carlsbad, CA) at 0.02% (v/v) for plate culture, and 0.06% (v/v) of 1,000 nm and 0.02% (v/v) of 100/20 nm LxB for stirred culture in the presence or absence of 10 ng/ml ultra pure LPS (*E. coli* 0111:B4; Invitrogen) for 24 h in plates or 9 h in stirred culture. For inhibition analyses using cytochalasin D (Sigma-Aldrich, Irvine, CA) apyrase (Sigma-Aldrich), N-acetylcysteine (NAC) and CA-074-Me (Calbiochem, Darmstadt, Germany), cells were treated with inhibitors 1 h prior to the stimulation at the concentrations indicated. For stimulation with ATP, BMDM were first treated with LPS for 6 h and subsequently cultured for 3 h in the presence of 1 mM ATP (Sigma-Aldrich) in stirred culture.

### Analyses of LxB associated with BMDM

BMDM (2.0 x 10^5^) in tissue culture plates were incubated with 0.02% fluorescenated LxB in the presence of LPS (10 ng/ml) for 24 h. After washing with ice-cold PBS, the cells were lysed with 1% SDS in PBS, and the fluorescence intensity of the lysate was measured using Spectra Max Gemini (Molecular Device, Downingtown, PA). The numbers of LxB were calculated according to the manufacturer’s data sheet, and the total volume and surface area of each size of LxB were adapted to the number of particles.

### Cytokine measurements and Western blotting

IL-1β was assessed by sandwich ELISA using biotin-conjugated rabbit polyclonal anti-mouse IL-1β (e-Bioscience, San Diego, CA) as detection Ab and anti-mouse IL-1β mAbs (B122) (BD Biosciences, San Diego, CA) as capture Ab. Recombinant mouse IL-1β (BD Biosciences) served as control. TNF-α production was assessed by Cytometric Bead Array (CBA) mouse inflammatory kit (BD Biosciences).

After stimulation of cells for 9 h in the stirred culture as described above, cell lysates for pro-IL-1β, pro-caspase-1 and caspase-1 and culture supernatants for IL-1β were subjected to Western blot analyses using goat polyclonal anti-mouse IL-1β (R&D Systems, Minneapolis, MN) and polyclonal rabbit anti-mouse caspase-1 p10 (Santa Cruz Biotechnology, Santa Cruz, CA) after diluting with the loading buffer.

### Microscopic analyses

Before stimulation, BMDM were incubated on poly-L-lysine-coated glass coverslips for 12 h. Cells were fixed in 1% formaldehyde after 24 h stimulation with fluoresceinated LxB and observed using a phase-contrast microscope. In order to detect the localization of Lamp-1 and LC3, BMDM from LC3-GFP knock-in mice were stimulated with LxB + LPS for 15–18 h and fixed with 1% formaldehyde followed by permeabilization with 0.01% saponin. After staining with anti-CD107a/Lamp-1 (1D4B) + biotin-labeled anti-rat IgG + avidin-Cy3 and anti-GFP (Life Technology, Grand Island, NY) + FITC labeled anti-rabbit IgG, specimens mounted with glycerin-PBS (1:1) containing 1% propylgallate (Wako Pure Chemicals, Osaka, Japan) were observed using a deconvolution microscope BX51-FL (Olympus, Tokyo, Japan) and imaging software (SlideBook; Intelligent Imaging Innovation, Denver, CO). In some cases, nuclei were stained with 1 µM of DAPI (Wako Pure Chemicals).

For TEM, cells on cover slips were fixed with 2.5% glutaraldehyde and post-fixed with 2% osmium (VIII) oxide. Ultrathin sections were observed under a Hitachi H-7000 electron microscope at the Center for Anatomical Studies and the Laboratory of Diagnostic Pathology, Graduate School of Medicine, Kyoto University.

### Annexin V and PI staining

BMDM stimulated with LxB + LPS in the stirred culture were washed with PBS once, and stained with FITC-conjugated annexin V and PI at various time points. After incubation on ice for 20min, cells were washed with Hanks’ Balanced Salt Solutions (HBSS) once and resuspended in 300 µl of HBSS for flow cytometric analysis. Staurosporine-induced cell death served as a positive control.

### Detection of lysosome rupture

Rupture of the lysosomes in BMDM was monitored using fluorescent cathepsin B substrate (Magic Red) and acridine orange (Immunochemistry Technologies, Bloomington, MN) in accordance with the manufacturer’s protocol by adding the reagents for the last 20 min of 18 h of culture with LPS + non-fluoresceinated LxB. Cells were observed using the BZ-8000 Biozero imaging device (Keyence, Osaka, Japan).

### Analyses of intracellular oxidative burst by flow cytometry

BMDM stimulated with non-fluoresceinated LxB beads in the presence or absence of LPS instirred culture for 1 h were incubated with 10 mM of dihydrorhodamine-123 (DHR-123) (Sigma-Aldrich) for 20 min as described previously [30]. Cells were acquired by flow cytometer (FACSCalibur, BD Biosciences), and data were analyzed using FlowJo software (Tree Star Inc., San Carlos, CA).

### Analyses of mitochondrial mass and membrane potential for respiratory activity

After stimulation for 6 h in the stirred culture, BMDM were treated with Mitotracker (Invitrogen) green (25 nM) and deep red (20 nM) to detect mitochondrial mass and membrane potential, respectively, and then analyzed by flow cytometer.

### Statistical analysis

Data are expressed as the mean and s.d. of triplicate cultures. Statistical significance was determined by the two-tailed Student’s *t*-test or multiple comparisons with Tukey’s multiple range test as indicated in the legends. All experiments were performed at least three times and representative results are shown.

## Results

### Vacuole formation and IL-1β production by various sizes of LxB

**Figure 1:**
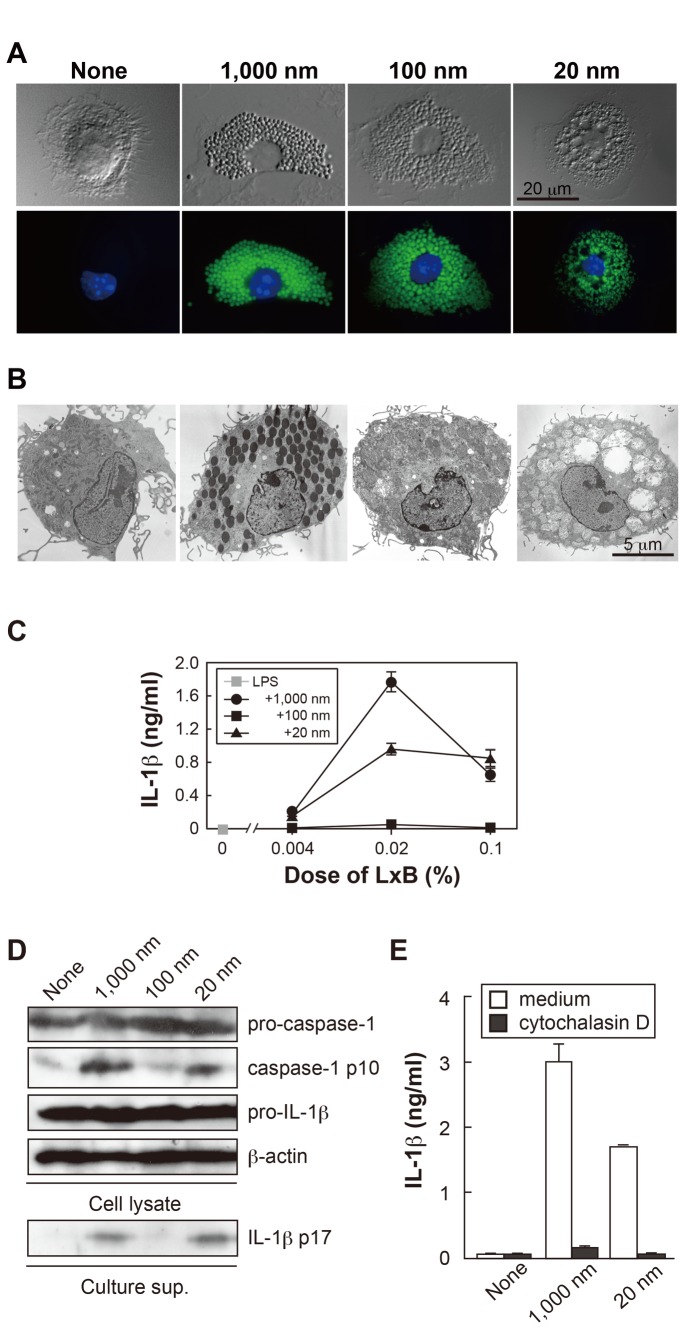
Morphological change and IL-1β production of BMDM after stimulation with size-defined LxB. (A) Uptake of various sizes of fluoresceinated LxB (0.02%) by BMDM (BALB/c) was observed 24 h after culture in the presence of LPS using phase-contrast (*upper*) and fluorescence (*lower*) microscopes. Scale bar, 20 µm. (B) BMDM were cultured with un-labeled LxB as in (A) and observed by TEM. (C) BMDM derived from BALB/c mice were cultured with different sizes of LxB (0.02%) at various concentrations in the presence of LPS for 24 h, and IL-1β production was then measured. (D) Production of pro-IL-1β, mature IL-1β (IL-1β p17), pro-caspase-1 and caspase-1 p10 was analyzed by Western blotting 9 h after stimulation with LxB (0.02%) + LPS. (E) BMDM were treated with cytochalasin D for 1 h prior to the stimulation in plate culture with LxB (0.02%) + LPS for 24 h, and IL-1β production was then assessed. Error bars represent s.d. of triplicate cultures within each group. All results are representative of at least 3 independent assays.

To prove the possibility that cells respond to different sizes of particles through distinct mechanisms and triggering pathways, we employed LxB with uniform diameters of 1,000 nm, 100 nm or 20 nm. Only 20 nm LxB resulted in the formation of vacuoles (Figure 1A). With transmission electron microscopy, LxB of 1,000 and 100 nm were shown to localize in the phagosomal vesicles (Figure 1B). On the other hand, 20 nm LxB were distributed at the rim of swollen/enlarged phagolysosomal vesicles (Figure 1A). Furthermore, these vesicles appeared to fuse with each other to be vacuolized (Figure 1B).

Interestingly, both 1,000 nm and 20 nm, but not 100 nm, LxB induced IL-1β production in the presence of LPS, although LPS alone did not (Figure 1C and 1D). IL-1β production by 1,000 and 20 nm LxB was also shown to depend on endocytosis (Figure 1E). The inability of 100 nm LxB to induce IL-1β production was due to the lack of active caspase-1 p10 (Figure 1D), even though 100 nm LxB was efficiently endocytosed by BMDC in terms of number, surface area and volume (Table SI). Of note it was that 30 and 300 nm LxB behaved like 20 and 1,000 nm LxB, respectively (data not shown). LPS-induced pro-IL-1β (Figure 1D) and TNF-α production (Figure S1A) were not affected by any size of LxB. The morphological changes described above and cytokine production patterns were also observed in the case of primary microglia cells (Figure S2).

### IL-1β production in the stirred culture of BMDM

**Figure 2:**
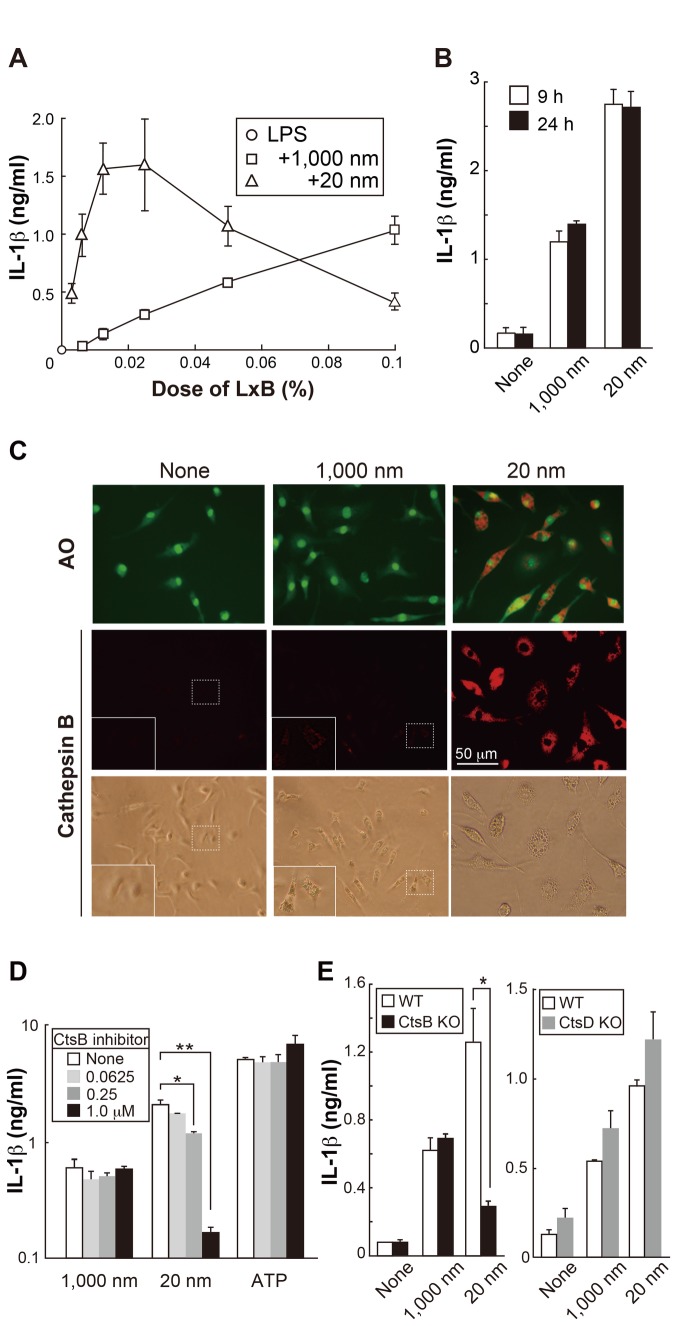
Contribution of cathepsin B pathway for IL-1β production in response to 20 nm LxB. (A) BMDM (BALB/c) (3 x 10^4^ cells) in 100 µl culture medium supplemented with LPS and various concentrations of LxB were cultured in a 1.5 ml microtube by stirring using a rotator in a CO_2_ incubator for 24 h, and IL-1β production was then assessed. (B) BMDM were stimulated with 0.06% 1,000 nm and 0.02% 20 nm LxB for 9 and 24 h, and IL-1β production was then assessed. (C) BMDM on coverslips were treated with acridine orange (AO) and fluorescent cathepsin B substrate for the last 20 min of 18 h culture with LxB (0.02%) + LPS. In some cases, the area with the white dashed line is enlarged and superimposed. (D) After treating with various doses of cathepsin B inhibitor (CA-074-Me) and vehicle (DMSO) for 1 h, BMDM were then stimulated with LxB + LPS in the stirred culture as in (B) for 9 h. *P < 0.005, **P < 0.003 (*t*-test). (E) Cathepsin B- and D-deficient and WT BMDM (C57BL/6) were stimulated as in (B) for 9 h. *P < 0.01 (*t*-test). Error bars represent s.d. of triplicate cultures within each group. All results are representative of at least 3 independent assays.

When LxB were used in plate culture, the larger LxB tended to settle faster than the smaller ones. Therefore, BMDM were stimulated in a stirred suspension culture with various doses of 1,000 nm and 20 nm LxB for 24 h. The results demonstrated that 20 nm LxB effectively induced a strong response at a lower dose, whereas the 1,000 nm LxB dose needed to be higher (Figure 2A). When cells were stimulated with 0.02% of 20 nm LxB and 0.06% of 1,000 nm LxB for 9 h and 24 h, responses were comparable between 9 and 24 h in each size of LxB (Figure 2B), indicating that a short period (9 h) of stimulation was enough in the stirred culture. Under such culture conditions, no IL-1β production was detected after stimulation with any size of LxB (Figure S3). In regards to cell damage from the stimulation, there was no difference in cell death between the 20 and 1,000 nm LxB at the end of the stirred culture for 9 h (Figure S4). Of interest was that cells stimulated with 100 nm LxB looked slightly but significantly less damaged than those 20 and 1,000 nm LxB, implying the relation with inability of IL-1β production by 100 nm LxB.

### IL-1β production by 20 nm LxB largely depends on cathepsin B leaked from ruptured phagolysosomes

Silica particles have been shown to rupture lysosomal organelles, leading to the release of cathepsin B into cytosol and subsequent NLRP3 activation followed by IL-1β production [4]. This study also reported that silica induces cathepsin B-dependent IL-1β production of BMDM, being accompanied by lysosome swelling, which is consistent with our observation using 20 nm LxB. Therefore, it is possible that IL-1β production by 20 nm LxB depends on cathepsin B. To confirm this possibility, we first examined the rupture of phagolysosomes and the localization of cathepsin B in the cytoplasm by staining with acridine orange (AO) and fluorescent cathepsin B substrate, respectively. As shown in Figure 2C, leakage of AO and active cathepsin B were observed in the cytoplasm only when BMDM were stimulated with 20 nm LxB. In the case of 1,000 nm LxB, a weak signal indicating a fluorescent cathepsin B substrate was observed in the phagolysosomes, but none in the cytoplasm, whereas BMDM treated with LPS alone showed hardly any active cathepsin activity.

In addition, cathepsin B inhibitor (CA-074-Me) decreased IL-1β induction in response to 20 nm, but not 1,000 nm, LxB and ATP (Figure 2D). Moreover, IL-1β production by 20 nm LxB was reduced in cathepsin B-, but not D-, deficient BMDM (Figure 2E), although the formation of vacuoles was still observed (Figure S5). These results indicate that cathepsin B is required for IL-1β production, but not vacuole formation, in response to 20 nm LxB.

### ATP *via* P2X_7_ receptor is involved in IL-1β production by 20 nm LxB

K^+^ efflux and NLRP3 activation are shown to be indispensable in IL-1β induction by small particles [12–14]. This activation was also required for the induction of IL-1β, but not TNF-α, in response to either size of LxB (Figures S6A and S6B).

**Figure 3:**
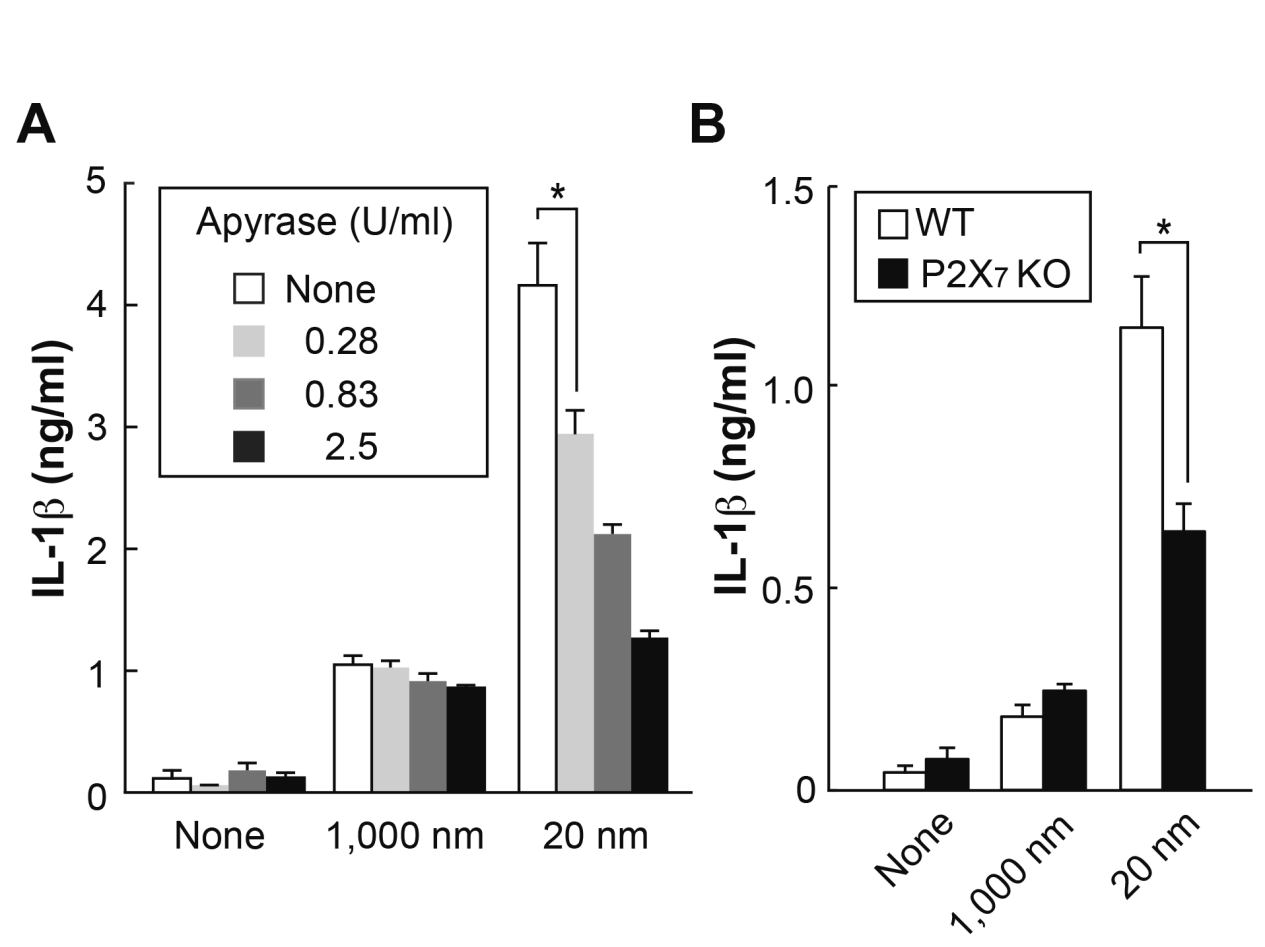
Contribution of ATP-P2X_7_-dependent pathway to IL-1β production in response to 20 nm LxB. (A) BMDM (BALB/c) were pre-treated with various doses of apyrase for 1 h prior to the stimulation with LxB (1,000 nm; 0.06%, 20 nm; 0.02%) + LPS for 9 h in stirred culture as in Figure 2D. *P < 0.01 (*t*-test). (B) BMDM from P2X_7_ deficient and WT mice (C57BL/6) were stimulated with LxB (1,000 nm; 0.06%, 20 nm; 0.02%) + LPS for 9 h in stirred culture as in Figure 2D as in Figure 2E. *P < 0.01 (*t*-test). Error bars represent s.d. of triplicate cultures within each group. All results are representative of at least 3 independent assays.

The sensing of ATP *via* the P2X_7_ receptor induces K^+^ efflux [26]. When apyrase, which accelerates the degradation of extracellular ATP, was added to the culture, IL-1β production was reduced only in response to 20, but not 1,000, nm LxB (Figure 3A). Furthermore, BMDM lacking the P2X_7_ receptor showed decreased IL-1β production only in response to 20 nm LxB (Figure 3B). These results suggest that the sensing of ATP by the P2X_7_ receptor is also involved in IL-1β production by 20 nm LxB.

### IL-1β production by 1,000 nm LxB relies on ROS

**Figure 4:**
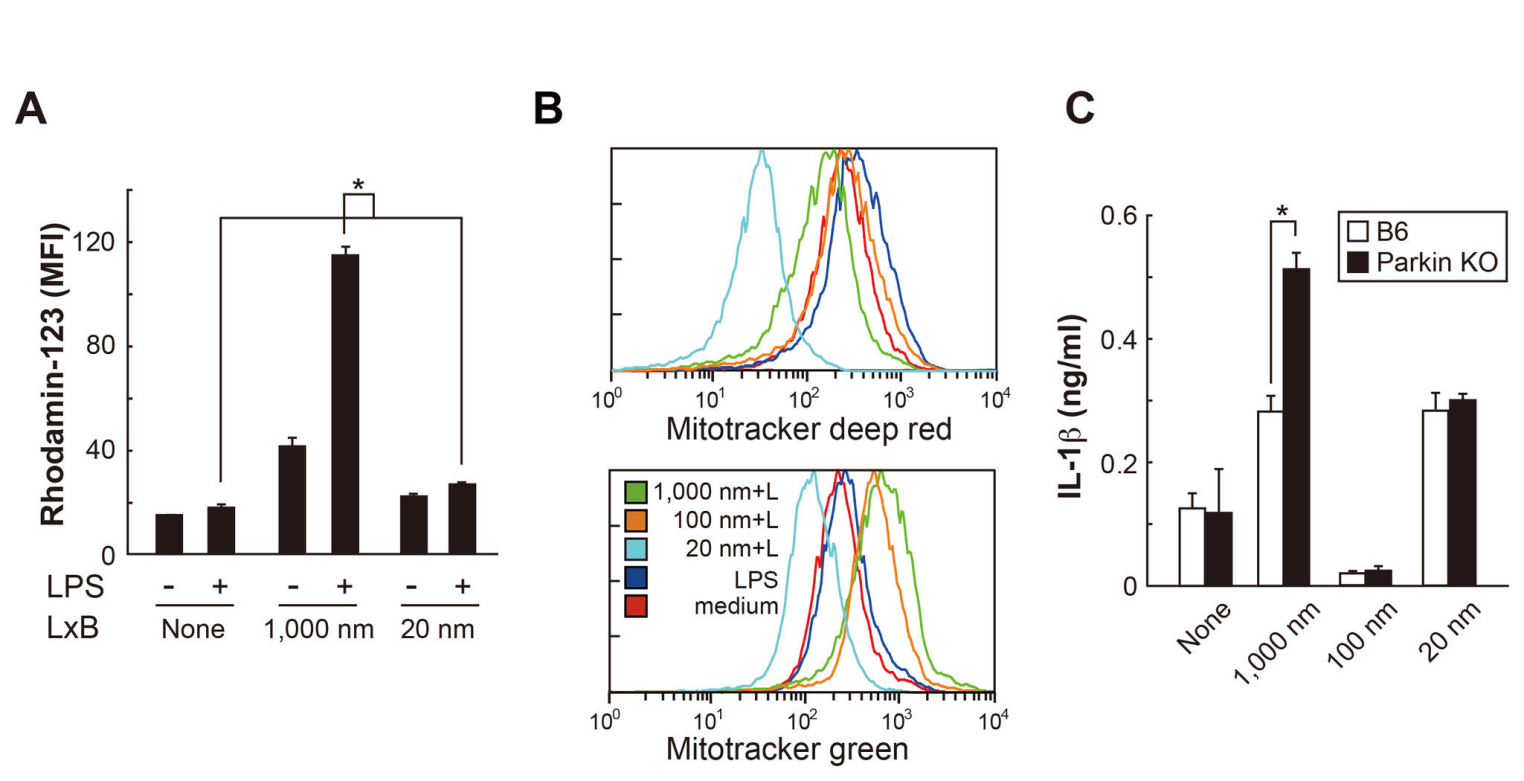
ROS generation and mitochondrial damage are caused by 1,000 nm LxB. (A) After stimulation with LxB (1,000 nm; 0.06%, 20 nm; 0.02%) ± LPS for 1 h in stirred culture, BMDM (BALB/c) were incubated with 10 µM of DHR-123 for 20 min. ROS activity was monitored by flow cytometer. *P < 0.01 (multiple comparison with Tukey’s test). (B) BMDM treated with Mitotracker green (25 nM) for mitochondrial mass and Mitotracker deep red (20 nM) for membrane potential for the last 20 min of 6 h culture with LxB (1,000 nm; 0.06%, 20 nm; 0.02%) + LPS (+L) were analyzed by flow cytometer. (C) BMDM from Parkin-deficient and WT mice (C57BL/6) were stimulated with LxB (1,000 nm; 0.06%, 20 nm; 0.02%) + LPS as in Figure 2E. *P < 0.01 (*t*-test). Error bars represent s.d. of triplicate cultures. All results are representative of at least 3 independent assays.

ROS induced by the particles was also shown to be another NLRP3 activator [15–17]. However, a robust intracellular ROS was induced only in response to 1,000 nm LxB, the response to 20 nm LxB being very weak (Figure 4A).

ROS production is known to follow the mitochondrial dysregulation induced by various types of stress. To examine this possibility, the membrane potential and total mass of mitochondria were assessed using Mitotracker deep red and Mitotracker green, respectively. LPS alone moderately increased membrane potential but did not affect mitochondrial mass, whereas 1,000 nm LxB moderately decreased membrane potential but markedly increased mitochondrial mass 6 h after the stimulation (Figure 4B). Such changes were detected with 1,000 nm LxB even in the absence of LPS (Figure S7). In contrast, mitochondrial mass and membrane potential were dramatically reduced in response to 20 nm LxB with (Figure 4B) and without LPS (Figure S7). On the other hand, 100 nm LxB induced a significant increase in mitochondrial mass regardless of the presence or absence of LPS, but the membrane potential was not significantly different than that of medium alone (Figure 4B and S7).

Damaged mitochondria are cleared up by proteasomes and mitophagy, autophagy specific to mitochondria [31,32], and this process is preceded by the E3 ubiquitin-protein ligase Parkin [33]. The lack of the PARK2 encoding Parkin gives rise to the accumulation of dysfunctional elongated mitochondria [34]. PARK2-deficient BMDM produced significantly higher amounts of IL-1β than wild type in response to 1,000 nm, but not 20 nm, LxB (Figure 4C). It is of note that PARK2-deficient cells did not produce IL-1β in response to 100 nm LxB nor to WT cells.

**Figure 5:**
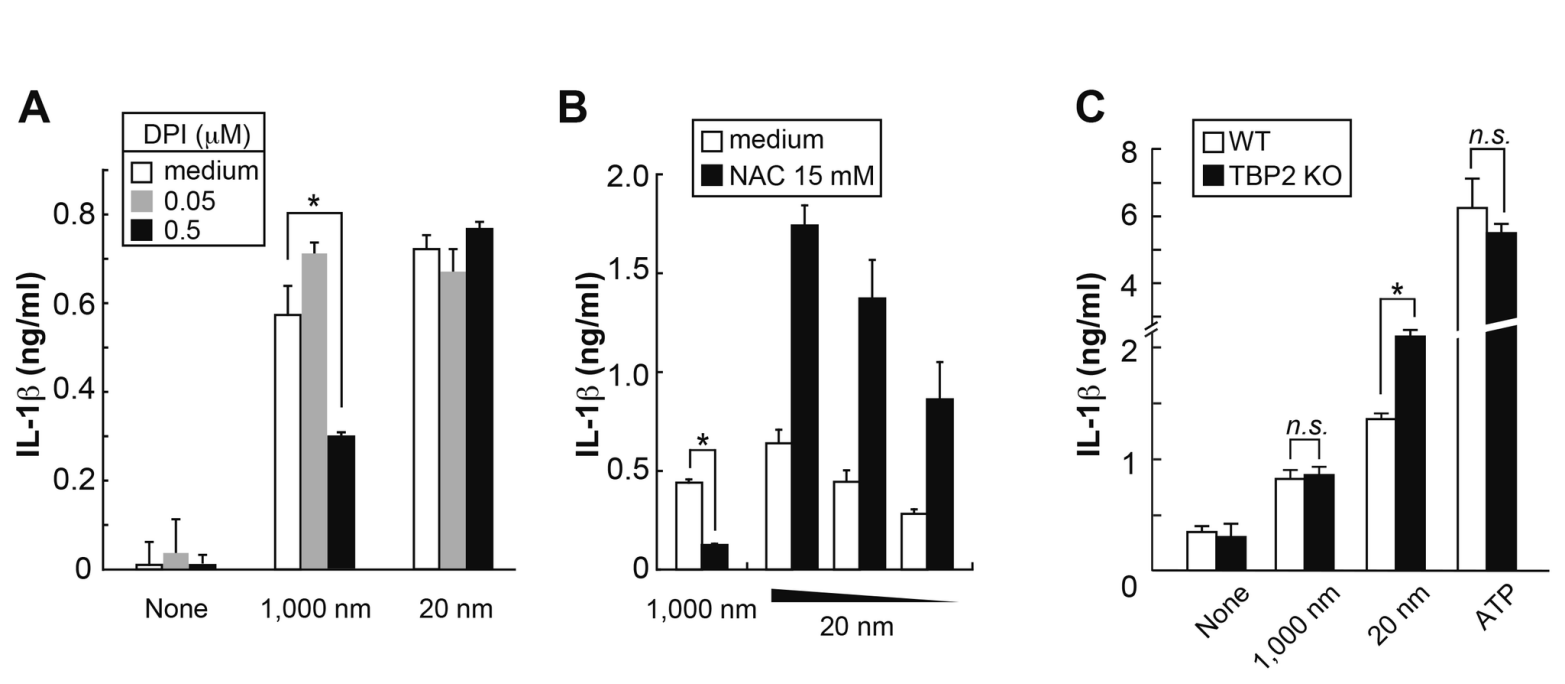
ROS mediates IL-1β production in response to 1,000 nm LxB. (A) BMDM (BALB/c) were stimulated with LPS for 3 h and subsequently treated with DPI for 1 h prior to the stimulation with LxB + LPS for 9 h. *P < 0.01 (*t*-test). (B) BMDM pre-treated with 15 mM of NAC for 1 h were stimulated with LPS + LxB for 9 h. (C) As in Figure 2E, TBP-2-deficient and WT BMDM (C57BL/6) were stimulated with LxB (1,000 nm; 0.06%, 20 nm; 0.02, 0.01 and 0.005%) + LPS. *P < 0.01 (*t*-test). All error bars are s.d. of triplicate cultures. All results are representative of at least 3 independent assays.

DPI, which irreversibly inactivates many redox-active proteins, has been reported to inhibit pro-IL-1β production *via* the suppression of NF-κB signaling [21], but has also been shown to inhibit NLRP3 activation through transcriptional repression [20]. When added after priming BMDM with LPS, DPI significantly inhibited IL-1β secretion in response to 1,000 nm LxB (Figure 5A). Consistent with this observation, anti-oxidant NAC also markedly reduced IL-1β production with 1,000 nm LxB (Figure 5B). In contrast, NAC augmented the response by 20 nm LxB at any dose tested (Figure 5B), while DPI did not show such augmentation. The inability of DPI to augment IL-1β production may be due to the incomplete suppression of ROS production by the DPI treatment following LPS stimulation.

TBP-2 has been reported to not only be an intracellular ROS sensor as an activator of NLRP3 inflammasomes [15] but also to be a negative regulator of a ROS scavenger thioredoxin [35,36]. When TBP-2-deficient BMDM were used, IL-1β production was significantly augmented in response to 20 nm, but not 1,000 nm, LxB compared with that of WT (Figure 5C), suggesting that a small amount of ROS produced from severely damaged mitochondria in response to 20 nm LxB interferes with IL-1β production *via* a cathepsin B-dependent pathway.

## Discussion

In this study we have dissected the molecular mechanisms responsible for IL-1β production depending on particle size using size-defined LxB. The results demonstrate that distinct signaling cascades are mainly utilized by different sized test particles in BMDM, although critical steps, such as the activation of NLRP3 inflammasome and K^+^ efflux, are shared in both cases. IL-1β production by nano-size particles relies on cathepsin B- and extracellular ATP-sensitive pathways, whereas that by micro-size particles relies on a ROS-mediated pathway. In addition, we observed that only nano-size LxB induced intracellular vacuole formation. Such size-dependent cellular responses have been hitherto concealed, possibly because the particles used in previous studies were not uniform in size.

Both 1,000 nm and 20 nm LxB were observed in the cells, although they appeared to be localized in the phagolysosomes and cytoplasm, respectively. Regarding the engulfment of small particles, it is known that, in some cases, small particles penetrate/diffuse across plasma membrane into cytosol [37]. However, cytochalasin D inhibited IL-1β production not only by 1,000 nm but also 20 nm LxB, indicating actin-mediated internalization, such as receptor-mediated phagocytosis and macropinocytosis. Therefore it is feasible that 20 nm LxB are somehow released from the endocytic vesicle to cytoplasm following their capture.

Both micro- and nano-size particles utilize the K^+^ efflux and NLRP3 inflammasome pathways to produce IL-1β. However, extracellular ATP and its receptor, P2X_7_ -- as a candidate inducer for K^+^ efflux, are involved only in response to 20 nm LxB. It has been reported that gene knockout of P2X_7_ doesn’t attenuate IL-1β production in response to silica, although P2X_7_ inhibitor (A740003) is moderately suppressive [38]. Larger sized silica particles tend to come into contact with and be internalized by BMDM in a short incubation culture, as a result of which a robust ROS production sequesters the effect of P2X_7_ deficiency by small sized silica. Therefore, the inconsistent results of the experiments using gene knockout mouse and inhibitor of P2X_7_ may depend on particle size. However, it remains plausible that other ATP- and P2X_7_-independent K^+^ efflux/NLRP3 activation pathways are involved in IL-1β production in response to 1,000 nm LxB.

Recently, long needle-like carbon nanotube and asbestos have been demonstrated to induce IL-1β secretion of human monocyte-derived macrophages in a ROS- and cathepsin B/P2X_7_ dependent manner through NLRP3 activation [6]. Compared with these needle-like materials, spherical carbon black with an average size of 14 nm is not effective for IL-1β production at the same concentration [6]. However, this may also be the case for size-dependent sedimentation between the test materials, although their shape should be taken into consideration. In a previous report, cathepsin B inhibitor (CA-074-Me) was shown to suppress the extracellular efflux of ATP and subsequent IL-1β production after stimulation with silica [38]. Therefore, it is possible that cathepsin B is an important molecule in the upstream part of the P2X_7_ pathway in response to 20 nm LxB.

Cathepsin B release into cytosol was observed only in response to 20 nm LxB, being consistent with the observation using amyloid-β [18]. This may relate to the phagolysosome swelling. Another possibility is that nano-size particles penetrate the vesicular membrane. It has been reported that amyloid-β induces cathepsin B-dependent IL-1β production of microglia cells [18]. Since amyloid-β peptide rapidly forms nano-size toxic oligomers [39], it is of particular interest that cells recognize such aggregated proteins and crystals as micro- and nano-size particles *in vivo*.

The involvement of ROS in IL-1β production was mainly in the case of 1,000 nm LxB. LxB of 1,000 nm resulted in the accumulation of weakly damaged mitochondria that sustain the activity of ROS generation. Parkin-deficient BMDM showed the up-regulation of IL-1β production by 1,000 nm LxB. Therefore, mitophagy seems to be involved in the regulation of IL-1β production [40]. ROS was not produced in any large amounts by 20 nm LxB, possibly due to severe damage and massive degradation, as shown by the decrease in membrane potential and mitochondrial mass. Of note is that the autophagosomal marker LC3 was associated with the membrane of the fused phagolysosomes and vacuoles after stimulation with 20 nm LxB (Figures S8A and S8B). Greater numbers of LC3^+^ puncta were also observed at an early time point (Figure S8B) using BMDM from LC3-GFP knock-in mouse, suggesting that such autophagic response is involved in the clearance of damaged mitochondria.

It is curious that the small amount of ROS produced in the course of the response to 20 nm LxB appeared to inhibit IL-1β production depending on cathepsin B, although the mechanism(s) involved are not clear at present. In the case of THP-1 (human acute monocytic leukemia cell line), 1,000 nm silica has been shown to first generate ROS, leading to subsequent rupture of the endosomes and cathepsin B release to cytosol [41]. This result seems to be at odds with that of our present study using LxB, since no apparent cathepsin B was observed in histochemistry in response to 1,000 nm LxB. Although the reason for this is unknown, this might be due to the cell type used or distinct surface properties.

An interesting observation in the present study was that 100 nm LxB were ineffective in inducing IL-1β production. This point should be the focus of future experiments in relation to the nature of this particular size of particle. On the other hand, Lunov et al. reported that amino (NH_2_)-modified, but not non-modified and carboxyl (COOH)-modified, 100 nm LxB induce IL-1β production from human Mø in ROS- and cathepsin B-dependent manners [42], suggesting the involvement of surface properties of particles in cellular responses. Since nano-size materials and crystals/aggregates have respective surface properties and cellular receptors, it is possible that material-specific cellular responses are induced. Nevertheless, our results indicating particle size-dependent signaling pathways (Figure S9) may provide some clues regarding the biological ‘nano-effect’ and help to set a new ‘nano-size’ standard in cell biology.

## Supporting Information

Figure S1 Effect of LxB on TNF-α production of BMDM. BMDM (BALB/c) were stimulated in culture plate with LxB (0.02%) and LPS, and TNF-α production was then analyzed by CBA 24 h later. Results are representative of 3 replicate experiments with triplicate cultures. Error bars represent s.d. within each group.
(TIF)

Figure S2 Cell morphology and cytokine production of primary microglia cells after the stimulation with LxB plus LPS. (A) Microglia cells from BALB/c mouse brain were cultured with fluorescenated LxB (0.02%) of different sizes and observed as in Figure 1A. (B) IL-1β production was determined by ELISA as in Figure 1E. Results are representative of 4 replicate experiments with triplicate cultures. Error bars represent s.d. within each group.
(TIF)

Figure S3 IL-1β production after stimulation with or without LPS in stirred culture. BMDM (BALB/c) were stimulated with LxB (1,000 nm; 0.06%, 20 nm; 0.02%) as in Figure 2B for 9 h in the presence or absence of LPS, and IL-1β production was then assessed. N.D.: not detected.
(TIF)

Figure S4 Effect of LxB on viability of BMDM. BMDM (BALB/c) were cultured with LPS, LxB (0.02%) or staurosporine (positive control) as in Figure 2B. After 9 h, cells were stained with FITC-conjugated annexin V and PI, and analyzed by flow cytometer. The double-positive cells were defined as dead cells. Results are representative of 3 replicate experiments.
(TIF)

Figure S5 Cell morphology of BMDM from cathepsin-deficient mice after the stimulation with 20 nm LxB plus LPS. BMDM from cathepsin B^-/-^ (*left panel*) and D^-/-^ (*right panel*) mice (C57BL/6) were cultured with 20 nm LxB (0.02%) and observed as in Figure 1A.
(TIF)

Figure S6 IL-1β production by both 1,000 and 20 nm LxB depend on K^+^ efflux and NLRP3. (A) BMDM (BALB/c) pre-treated in medium supplemented with KCl or NaCl (75 mM each) for 3 h prior to the stimulation with 1,000 and 20 nm LxB (1,000 nm; 0.06%, 20 nm; 0.02%) in the presence of LPS for 9 h in stirred culture, and IL-1β production was then assessed. (B) In the presence of LPS, NLRP3-deficient and WT (C57BL/6) BMDM were stimulated with 1,000 and 20 nm LxB (1,000 nm; 0.06%, 20 nm; 0.02%) for 9 h or with ATP (1 mM) for the last 3 h in stirred culture, and IL-1β and TNF-α production was then analyzed. Results are representative of 3 replicate experiments with triplicate cultures. Error bars represent s.d. within each group.
(TIF)

Figure S7 Mitochondrial mass and membrane potential after stimulation with LxB. BMDM (BALB/c) were cultured with either LxBs (1,000 nm or 20 nm) or LPS alone, followed by the treatment with Mitotracker for 20 min as in Figure 4B, and the cells were then analyzed.
(TIF)

Figure S8 Formation of GFP-LC3^+^ vacuole after stimulation with 20 nm LxB. (A) As in Figure 1A, BMDM from LC3-GFP knock-in mice (C57BL/6) were cultured with 1,000 and 20 nm LxB (0.02%) for 18 h and stained with anti-Lamp-1 (*red*) and anti-GFP (*green*). For 20 nm LxB, two representative pictures of vacuolization are shown. (B) At 8 h after stimulation as in (A), BMDM were stained with anti-GFP (*left panels*), and the number of LC3-GFP^+^ puncta per cell was counted (*right panel*). *P < 0.05 (Tukey’s test). Error bars represent s.d. of 100 cells within each group.
(TIF)

Figure S9 Model for the different pathways of IL-1β induction by various sizes of LxBs. LxBs (1,000, 100 and 20 nm in diameter) that are endocytosed by macrophages cause mitochondria damage. Mitochondria damaged by 1.000 nm LxB seem to be cleared by a Parkin-dependent pathway, but not efficiently, leading to an accumulation of damaged mitochondria, production of ROS and activation of NLRP3 inflammasomes. On the other hand, 20 nm LxB cause rupture of the endosomes, leading to the release of cathepsin B (and possibly together with 20 nm LxB) into cytosol, followed by the activation of NLRP3 inflammasomes. In this case, damaged mitochondria may be effectively cleared by LC3^+^-macroautophagosomes or another unknown mechanism, resulting in no IL-1β production. LxB of 20 nm also cause release of ATP, possibly activating the P2X_7_-NLRP3 pathway.
(TIF)

Table S1 Number, surface area and volume of LxBs associated with BMDM. (TIF)

## References

[1] Jiang W, Kim BY, Rutka JT, Chan WC (2008) Nanoparticle-mediated cellular response is size-dependent. Nat Nanotechnol 3: 145-150. doi:10.1038/nnano.2008.30. PubMed: 18654486. 18654486 10.1038/nnano.2008.30

[2] Kumar H, Kawai T, Akira S (2011) Pathogen recognition by the innate immune system. Int Rev Immunol 30: 16-34. doi:10.3109/08830185.2010.529976. PubMed: 21235323. 21235323 10.3109/08830185.2010.529976

[3] Schroder K, Tschopp J (2010) The inflammasomes. Cell 140: 821-832. doi:10.1016/j.cell.2010.01.040. PubMed: 20303873. 20303873 10.1016/j.cell.2010.01.040

[4] Hornung V, Bauernfeind F, Halle A, Samstad EO, Kono H et al. (2008) Silica crystals and aluminum salts activate the NALP3 inflammasome through phagosomal destabilization. Nat Immunol 9: 847-856. doi:10.1038/ni.1631. PubMed: 18604214. 18604214 10.1038/ni.1631 PMC2834784

[5] Dostert C, Pétrilli V, Van Bruggen R, Steele C, Mossman BT et al. (2008) Innate immune activation through Nalp3 inflammasome sensing of asbestos and silica. Science 320: 674-677. doi:10.1126/science.1156995. PubMed: 18403674. 18403674 10.1126/science.1156995 PMC2396588

[6] Palomäki J, Välimäki E, Sund J, Vippola M, Clausen PA et al. (2011) Long, needle-like carbon nanotubes and asbestos activate the NLRP3 inflammasome through a similar mechanism 5 Nano: Association of Caribbean States pp. 6861-6870. PubMed: 21800904. 10.1021/nn200595c 21800904

[7] Martinon F, Pétrilli V, Mayor A, Tardivel A, Tschopp J (2006) Gout-associated uric acid crystals activate the NALP3 inflammasome. Nature 440: 237-241. doi:10.1038/nature04516. PubMed: 16407889. 16407889 10.1038/nature04516

[8] Rajamäki K, Lappalainen J, Oörni K, Välimäki E, Matikainen S et al. (2010) Cholesterol crystals activate the NLRP3 inflammasome in human macrophages: a novel link between cholesterol metabolism and inflammation. PLOS ONE 5: e11765. doi:10.1371/journal.pone.0011765. PubMed: 20668705. 20668705 10.1371/journal.pone.0011765 PMC2909263

[9] Duewell P, Kono H, Rayner KJ, Sirois CM, Vladimer G et al. (2010) NLRP3 inflammasomes are required for atherogenesis and activated by cholesterol crystals. Nature 464: 1357-1361. doi:10.1038/nature08938. PubMed: 20428172. 20428172 10.1038/nature08938 PMC2946640

[10] Masters SL, Dunne A, Subramanian SL, Hull RL, Tannahill GM et al. (2010) Activation of the NLRP3 inflammasome by islet amyloid polypeptide provides a mechanism for enhanced IL-1β in type 2 diabetes. Nat Immunol 11: 897-904. doi:10.1038/ni.1935. PubMed: 20835230. 20835230 10.1038/ni.1935 PMC3103663

[11] Niemi K, Teirilä L, Lappalainen J, Rajamäki K, Baumann MH et al. (2011) Serum amyloid A activates the NLRP3 inflammasome via P2X_7_ receptor and a cathepsin B-sensitive pathway. J Immunol 186: 6119-6128. doi:10.4049/jimmunol.1002843. PubMed: 21508263. 21508263 10.4049/jimmunol.1002843

[12] Pétrilli V, Papin S, Dostert C, Mayor A, Martinon F et al. (2007) Activation of the NALP3 inflammasome is triggered by low intracellular potassium concentration. Cell Death Differ 14: 1583-1589. doi:10.1038/sj.cdd.4402195. PubMed: 17599094. 17599094 10.1038/sj.cdd.4402195

[13] Cassel SL, Eisenbarth SC, Iyer SS, Sadler JJ, Colegio OR, et al. (2008) The Nalp3 inflammasome is essential for the development of silicosis. Proc. Natl. U.S.A: Acad. Sci 105: 9035-9040

[14] Eisenbarth SC Colegio OR, OConnor W, Sutterwala FS, Flavell RA (2008) Crucial role for the Nalp3 inflammasome in the immunostimulatory properties of aluminium adjuvants. Nature 453: 1122-1126. 18496530 10.1038/nature06939 PMC4804622

[15] Zhou R, Tardivel A, Thorens B, Choi I, Tschopp J (2010) Thioredoxin-interacting protein links oxidative stress to inflammasome activation. Nat Immunol 11: 136-140. doi:10.1038/ni.1831. PubMed: 20023662. 20023662 10.1038/ni.1831

[16] Zhou R, Yazdi AS, Menu P, Tschopp J (2011) A role for mitochondria in NLRP3 inflammasome activation. Nature 469: 221-225. doi:10.1038/nature09663. PubMed: 21124315. 21124315 10.1038/nature09663

[17] Wen H, Gris D, Lei Y, Jha S, Zhang L et al. (2011) Fatty acid-induced NLRP3-ASC inflammasome activation interferes with insulin signaling. Nat Immunol 12: 408-415. doi:10.1038/ni.2022. PubMed: 21478880. 21478880 10.1038/ni.2022 PMC4090391

[18] Halle A, Hornung V, Petzold GC, Stewart CR, Monks BG et al. (2008) The NALP3 inflammasome is involved in the innate immune response to amyloid-β. Nat Immunol 9: 857-865. doi:10.1038/ni.1636. PubMed: 18604209. 18604209 10.1038/ni.1636 PMC3101478

[19] Dostert C, Guarda G, Romero JF, Menu P, Gross O et al. (2009) Malarial hemozoin is a Nalp3 inflammasome activating danger signal. PLOS ONE 4: e6510. doi:10.1371/journal.pone.0006510. PubMed: 19652710. 19652710 10.1371/journal.pone.0006510 PMC2714977

[20] Bauernfeind F, Bartok E, Rieger A, Franchi L, Núñez G et al. (2011) Cutting edge: Reactive oxygen species inhibitors block priming, but not activation, of the NLRP3 inflammasome. J Immunol 187: 613-617. doi:10.4049/jimmunol.1100613. PubMed: 21677136. 21677136 10.4049/jimmunol.1100613 PMC3131480

[21] van de Veerdonk FL, Smeekens SP, Joosten LA, Kullberg BJ, Dinarello CA et al. (2010) Reactive oxygen species-independent activation of the IL-1β inflammasome in cells from patients with chronic granulomatous disease. Proc Natl Acad Sci U S A 107: 3030-3033. doi:10.1073/pnas.0914795107. PubMed: 20133696. 20133696 10.1073/pnas.0914795107 PMC2840365

[22] Bruchard M, Mignot G, Derangère V, Chalmin F, Chevriaux A et al. (2013) Chemotherapy-triggered cathepsin B release in myeloid-derived suppressor cells activates the Nlrp3 inflammasome and promotes tumor growth. Nat Med 19: 57-64. PubMed: 23202296. 23202296 10.1038/nm.2999

[23] Mizushima N, Yamamoto A, Matsui M, Yoshimori T, Ohsumi Y (2004) In vivo analysis of autophagy in response to nutrient starvation using transgenic mice expressing a fluorescent autophagosome marker. Mol Biol Cell 15: 1101-1111. PubMed: 14699058. 14699058 10.1091/mbc.E03-09-0704 PMC363084

[24] Kitao Y, Imai Y, Ozawa K, Kataoka A, Ikeda T et al. (2007) Pael receptor induces death of dopaminergic neurons in the substantia nigra via endoplasmic reticulum stress and dopamine toxicity, which is enhanced under condition of parkin inactivation. Hum Mol Genet 16: 50-60. doi:10.1093/hmg/ddm018. PubMed: 17116640. 17116640 10.1093/hmg/ddl439

[25] Oka S, Liu W, Masutani H, Hirata H, Shinkai Y et al. (2006) Impaired fatty acid utilization in thioredoxin binding protein-2 (TBP-2)-deficient mice: a unique animal model of Reye syndrome. FASEB J 20: 121-123. PubMed: 16254043. 16254043 10.1096/fj.05-4439fje

[26] Mariathasan S, Weiss DS, Newton K, McBride J, O’Rourke K et al. (2006) Cryopyrin activates the inflammasome in response to toxins and ATP. Nature 440: 228-232. doi:10.1038/nature04515. PubMed: 16407890. 16407890 10.1038/nature04515

[27] Deussing J, Roth W, Saftig P, Peters C, Ploegh HL et al. (1998) Cathepsins B and D are dispensable for major histocompatibility complex class II-mediated antigen presentation. Proc Natl Acad Sci U S A 95: 4516-4521. doi:10.1073/pnas.95.8.4516. PubMed: 9539769. 9539769 10.1073/pnas.95.8.4516 PMC22521

[28] Saftig P, Hetman M, Schmahl W, Weber K, Heine L et al. (1995) Mice deficient for the lysosomal proteinase cathepsin D exhibit progressive atrophy of the intestinal mucosa and profound destruction of lymphoid cells. EMBO J 14: 3599-3608. PubMed: 7641679. 7641679 10.1002/j.1460-2075.1995.tb00029.x PMC394433

[29] Nakajima K, Tsuzaki N, Shimojo M, Hamanoue M, Kohsaka S (1992) Microglia isolated from rat brain secrete a urokinase-type plasminogen activator. Brain Res 577: 285-292. doi:10.1016/0006-8993(92)90285-H. PubMed: 1376634. 1376634 10.1016/0006-8993(92)90285-h

[30] Takahara K, Tokieda S, Nagaoka K, Takeda T, Kimura Y et al. (2011) C-type lectin SIGNR1 enhances cellular oxidative burst response against *C. albicans* in cooperation with Dectin-1. Eur J Immunol 41: 1435-1444. doi:10.1002/eji.200940188. PubMed: 21400494. 21400494 10.1002/eji.200940188

[31] Yoshii SR, Kishi C, Ishihara N, Mizushima N (2011) Parkin mediates proteasome-dependent protein degradation and rupture of the outer mitochondrial membrane. J Biol Chem 286: 19630-19640. doi:10.1074/jbc.M110.209338. PubMed: 21454557. 21454557 10.1074/jbc.M110.209338 PMC3103342

[32] Youle RJ, Narendra DP (2011) Mechanisms of mitophagy. Nat Rev Mol Cell Biol 12: 9-14. doi:10.1038/nrm3028. PubMed: 21179058. 21179058 10.1038/nrm3028 PMC4780047

[33] Kitada T, Asakawa S, Hattori N, Matsumine H, Yamamura Y et al. (1998) Mutations in the parkin gene cause autosomal recessive juvenile parkinsonism. Nature 392: 605-608. doi:10.1038/33416. PubMed: 9560156. 9560156 10.1038/33416

[34] Ziviani E, Tao RN, Whitworth AJ (2010) Drosophila parkin requires PINK1 for mitochondrial translocation and ubiquitinates mitofusin. Proc Natl Acad Sci U S A 107: 5018-5023. doi:10.1073/pnas.0913485107. PubMed: 20194754. 20194754 10.1073/pnas.0913485107 PMC2841909

[35] Marks PA (2006) Thioredoxin in cancer--role of histone deacetylase inhibitors. Semin Cancer Biol 16: 436-443. doi:10.1016/j.semcancer.2006.09.005. PubMed: 17095247. 17095247 10.1016/j.semcancer.2006.09.005 PMC2766865

[36] Hernandez-Cuellar E, Tsuchiya K, Hara H, Fang R, Sakai S et al. (2012) Cutting Edge: Nitric Oxide Inhibits the NLRP3 Inflammasome. J Immunol 189: 5113-5117. doi:10.4049/jimmunol.1202479. PubMed: 23100513. 23100513 10.4049/jimmunol.1202479

[37] Verma A, Uzun O, Hu Y, Hu Y, Han HS et al. (2008) Surface-structure-regulated cell-membrane penetration by monolayer-protected nanoparticles. Nat Mater 7: 588-595. doi:10.1038/nmat2202. PubMed: 18500347. 18500347 10.1038/nmat2202 PMC2684029

[38] Riteau N, Baron L, Villeret B, Guillou N, Savigny F et al. (2012) ATP release and purinergic signaling: a common pathway for particle-mediated inflammasome activation. Cell Death Dis 3: e403. doi:10.1038/cddis.2012.144. PubMed: 23059822. 23059822 10.1038/cddis.2012.144 PMC3481132

[39] Lee J, Culyba EK, Powers ET, Kelly JW (2011) Amyloid-β forms fibrils by nucleated conformational conversion of oligomers. Nat Chem Biol 7: 602-609. doi:10.1038/nchembio.624. PubMed: 21804535. 21804535 10.1038/nchembio.624 PMC3158298

[40] Lupfer C, Thomas PG, Anand PK, Vogel P, Milasta S et al. (2013) Receptor interacting protein kinase 2-mediated mitophagy regulates inflammasome activation during virus infection. Nat Immunol, 14: 480–8. PubMed: 23525089. 23525089 10.1038/ni.2563 PMC3631456

[41] Morishige T, Yoshioka Y, Inakura H, Tanabe A, Yao X et al. (2010) The effect of surface modification of amorphous silica particles on NLRP3 inflammasome mediated IL-1β production, ROS production and endosomal rupture. Biomaterials 31: 6833-6842. doi:10.1016/j.biomaterials.2010.05.036. PubMed: 20561679. 20561679 10.1016/j.biomaterials.2010.05.036

[42] Lunov O, Syrovets T, Loos C, Nienhaus GU, Mailänder V et al. (2011) Amino-functionalized polystyrene nanoparticles activate the NLRP3 inflammasome in human macrophages 5 Nano: Association of Caribbean States pp. 9648-9657. PubMed: 22111911. 10.1021/nn203596e 22111911

