# Supplementary figures and images for: Particle Size of Latex Beads Dictates IL-1β Production Mechanism
Source: PLoS One. 2013 Jul 9;8(7):e68499. doi: 10.1371/journal.pone.0068499 (PMC3711474; doi:10.1371/journal.pone.0068499)

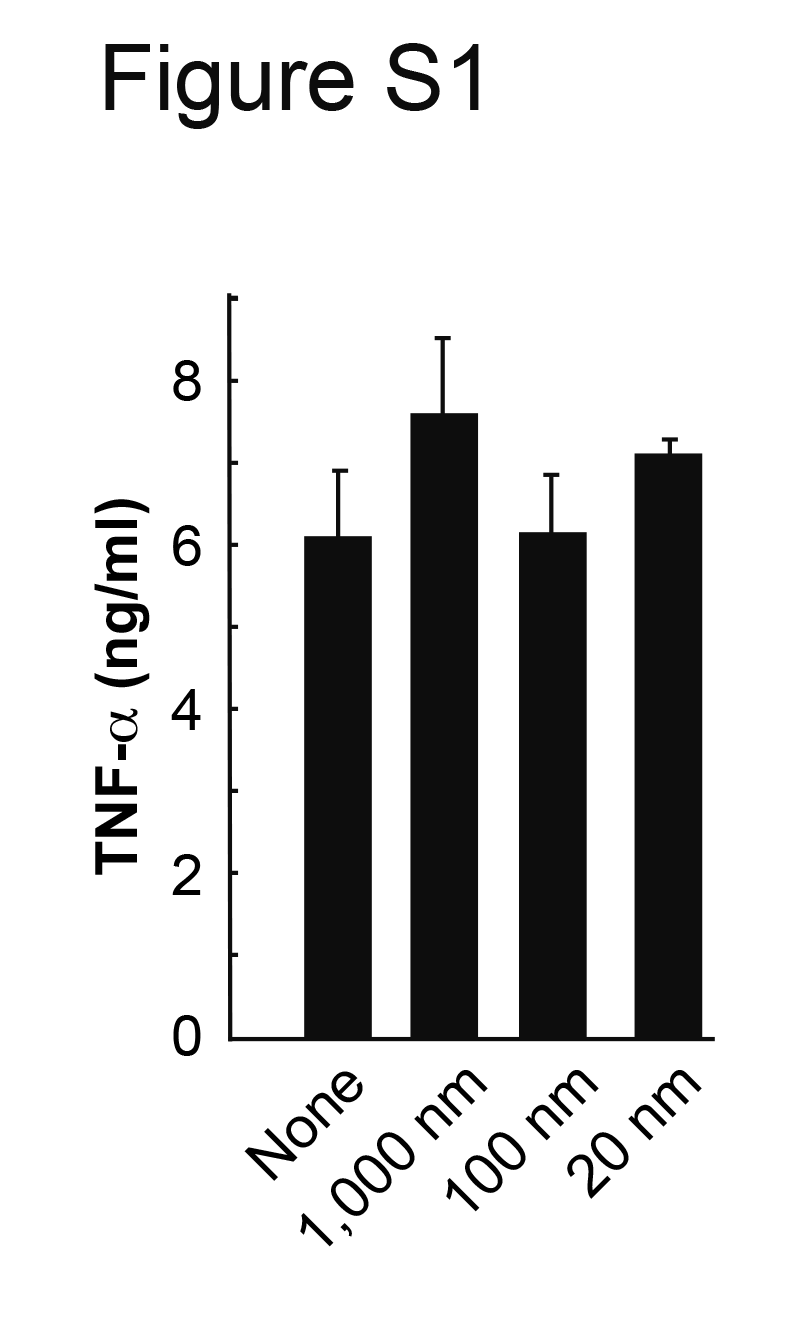

Supplement: Figure S1 — BMDM (BALB/c) were stimulated in culture plate with LxB (0.02%) and LPS, and TNF-α production was then analyzed by CBA 24 h later. Results are representative of 3 replicate experiments with triplicate cultures. Error bars represent s.d. within each group. (TIF) [file pone.0068499.s001.tif]

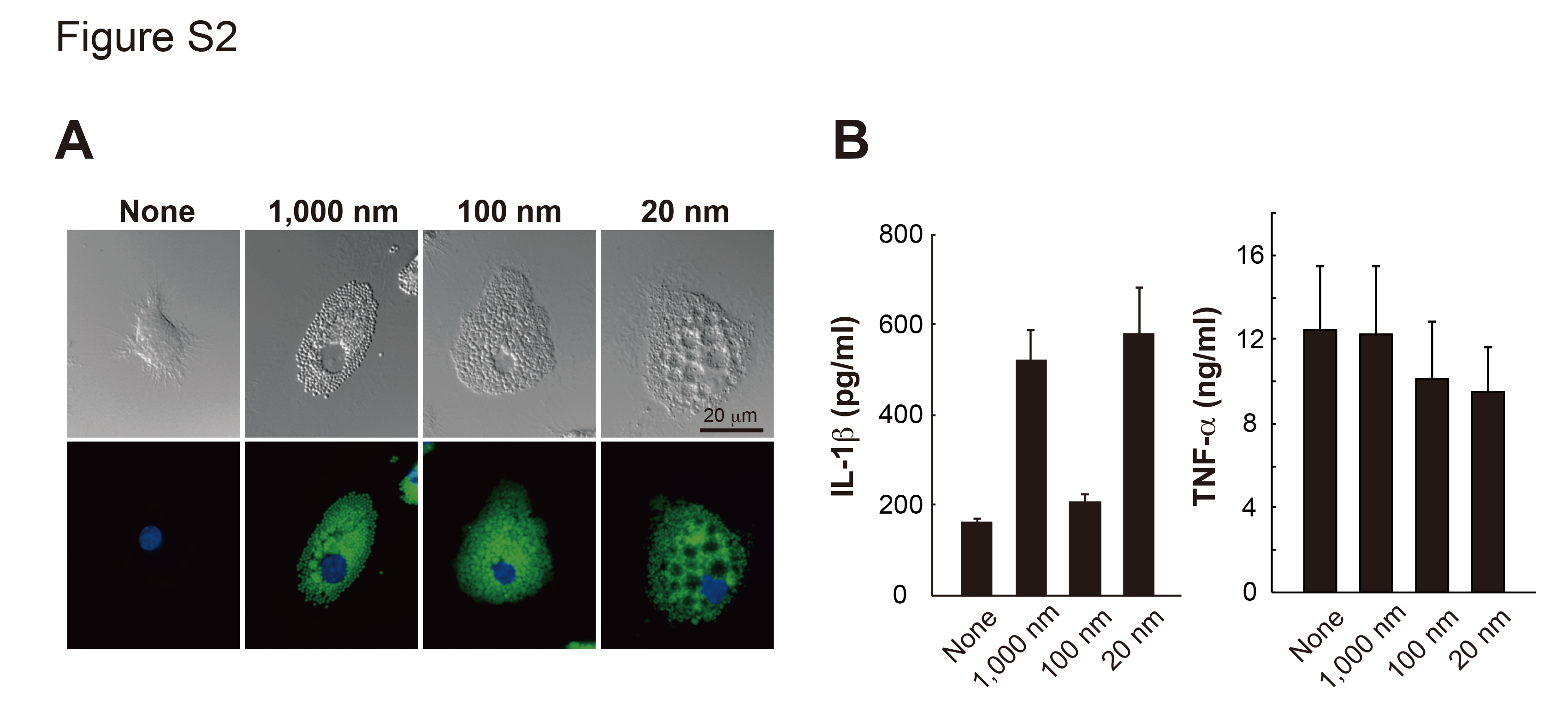

Supplement: Figure S2 — (A) Microglia cells from BALB/c mouse brain were cultured with fluorescenated LxB (0.02%) of different sizes and observed as in Figure 1A. (B) IL-1β production was determined by ELISA as in Figure 1E. Results are representative of 4 replicate experiments with triplicate cultures. Error bars represent s.d. within each group. (TIF) [file pone.0068499.s002.tif]

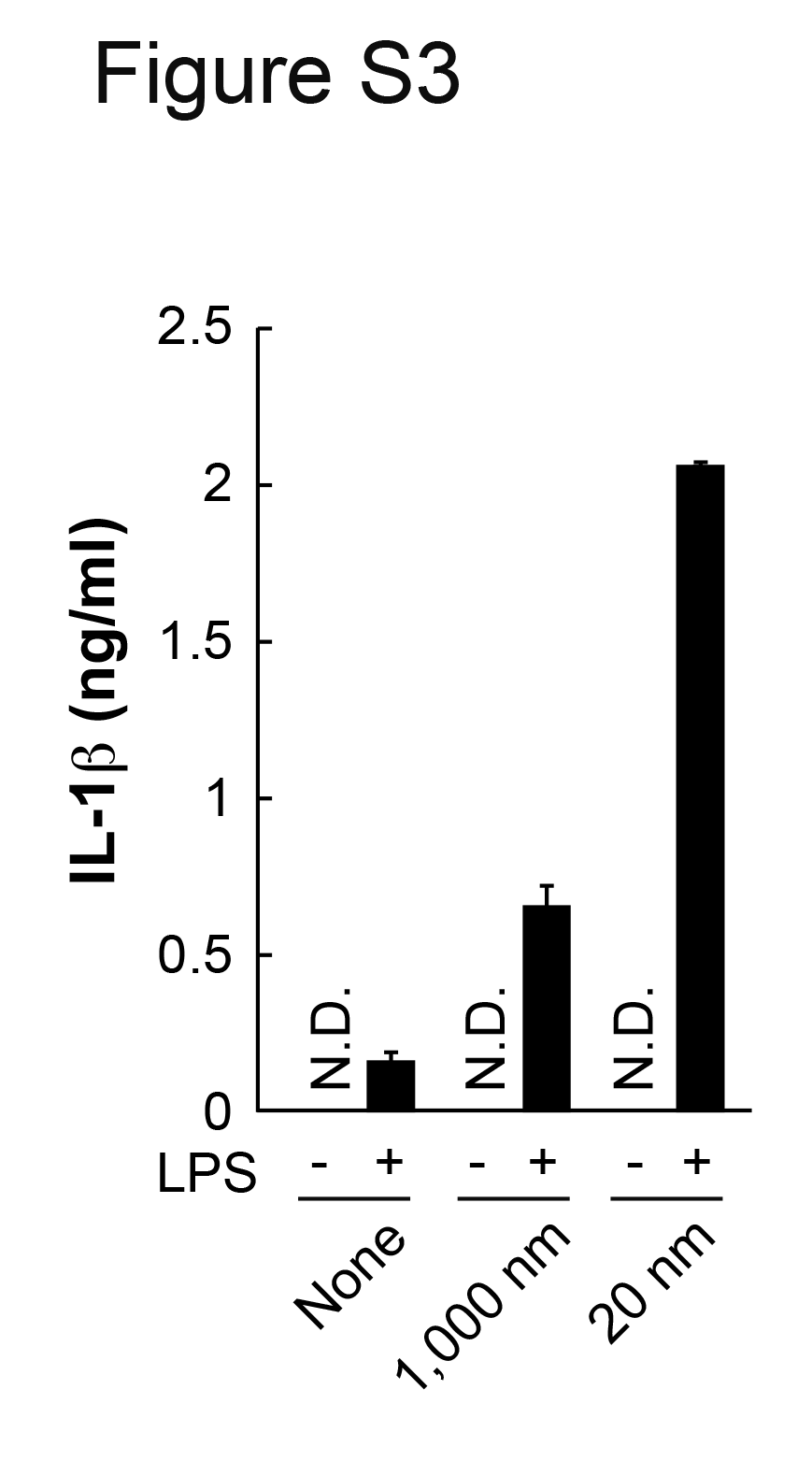

Supplement: Figure S3 — BMDM (BALB/c) were stimulated with LxB (1,000 nm; 0.06%, 20 nm; 0.02%) as in Figure 2B for 9 h in the presence or absence of LPS, and IL-1β production was then assessed. N.D.: not detected. (TIF) [file pone.0068499.s003.tif]

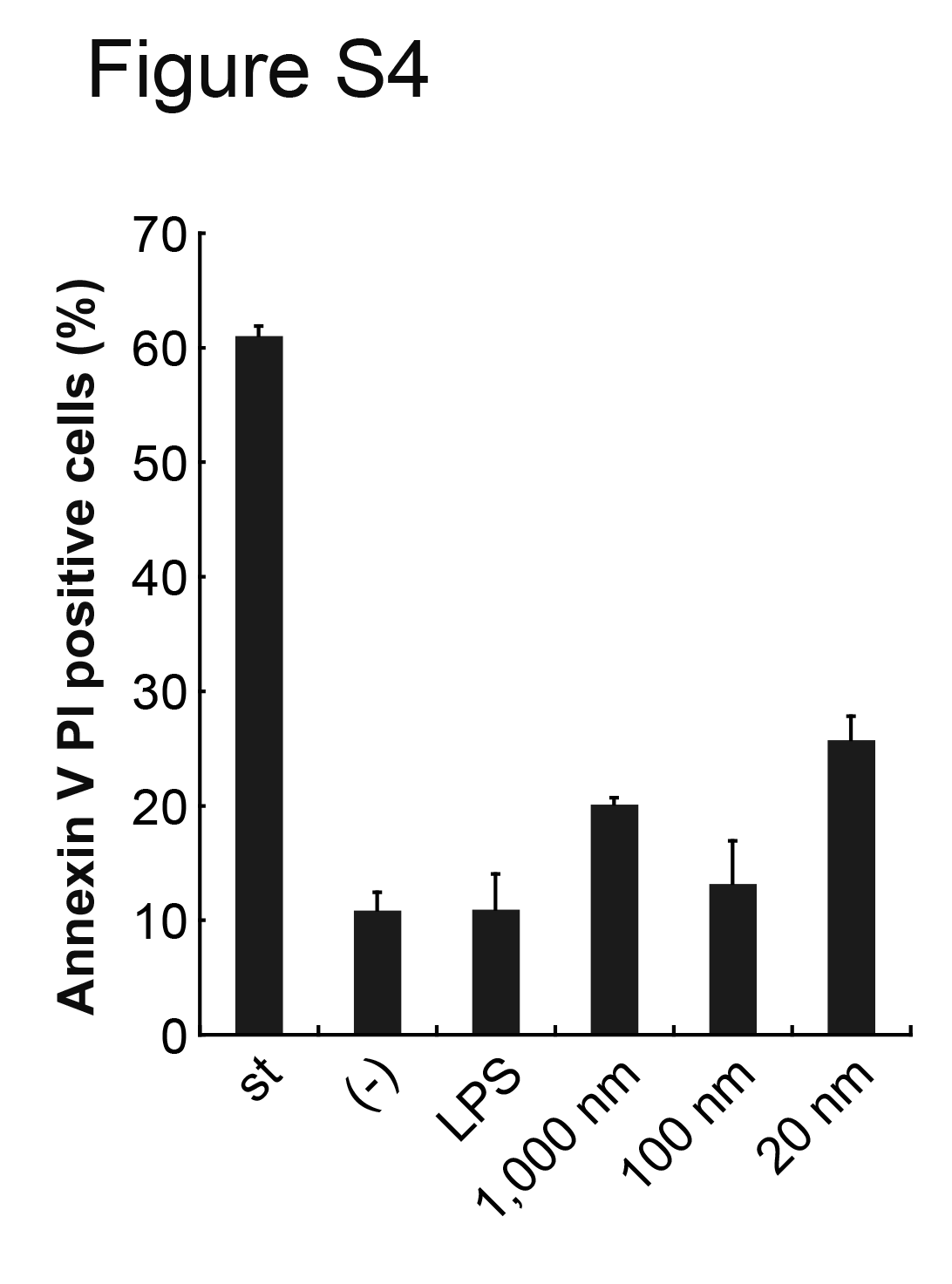

Supplement: Figure S4 — BMDM (BALB/c) were cultured with LPS, LxB (0.02%) or staurosporine (positive control) as in Figure 2B. After 9 h, cells were stained with FITC-conjugated annexin V and PI, and analyzed by flow cytometer. The double-positive cells were defined as dead cells. Results are representative of 3 replicate experiments. (TIF) [file pone.0068499.s004.tif]

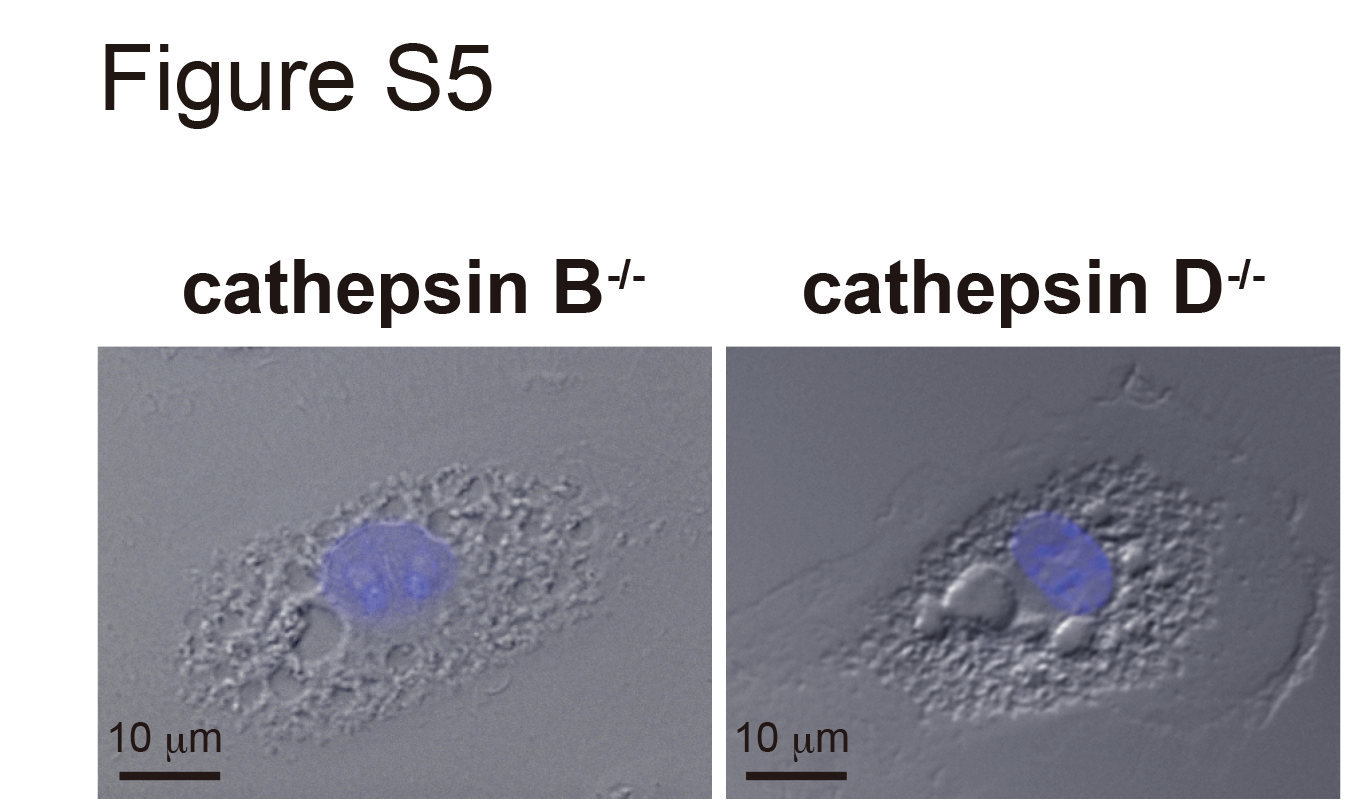

Supplement: Figure S5 — BMDM from cathepsin B-/- (left panel) and D-/- (right panel) mice (C57BL/6) were cultured with 20 nm LxB (0.02%) and observed as in Figure 1A. (TIF) [file pone.0068499.s005.tif]

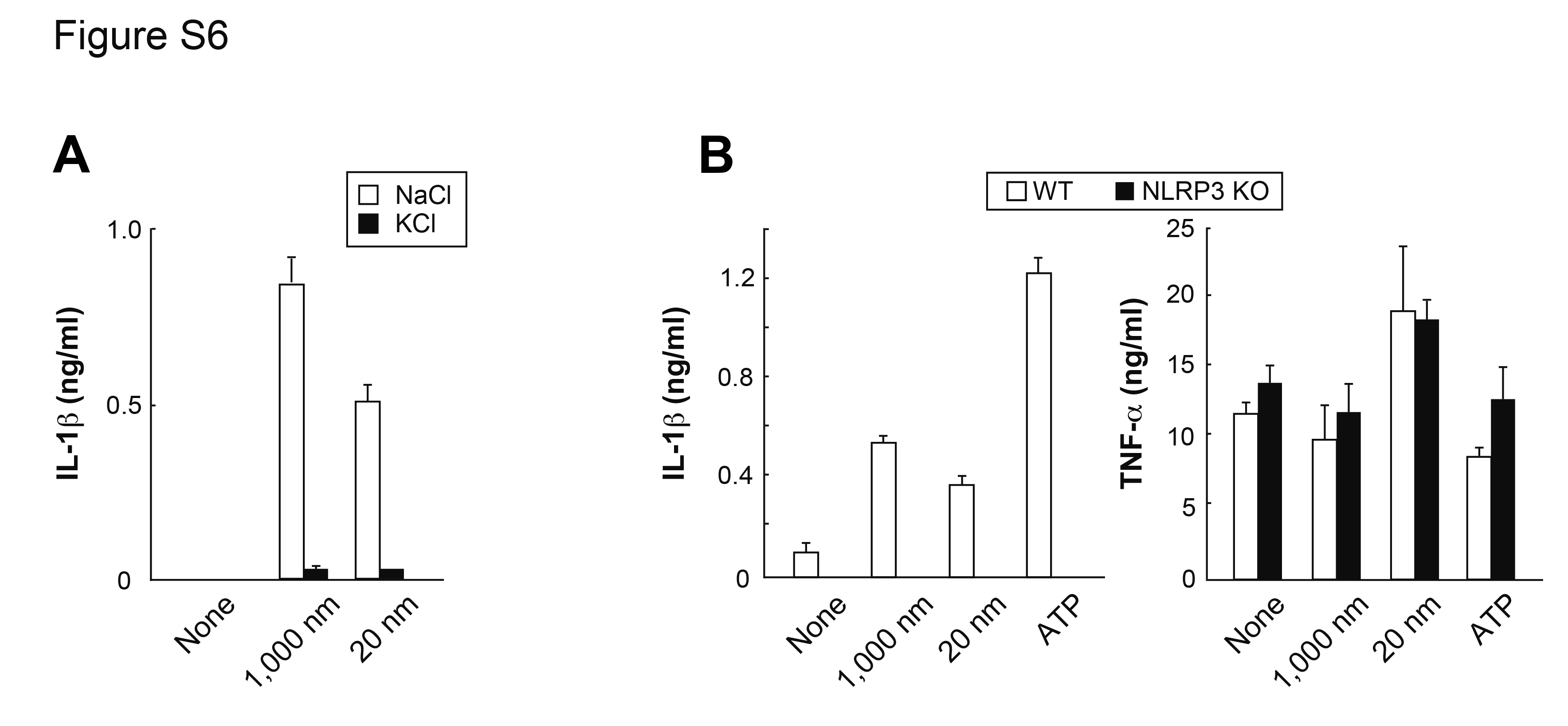

Supplement: Figure S6 — (A) BMDM (BALB/c) pre-treated in medium supplemented with KCl or NaCl (75 mM each) for 3 h prior to the stimulation with 1,000 and 20 nm LxB (1,000 nm; 0.06%, 20 nm; 0.02%) in the presence of LPS for 9 h in stirred culture, and IL-1β production was then assessed. (B) In the presence of LPS, NLRP3-deficient and WT (C57BL/6) BMDM were stimulated with 1,000 and 20 nm LxB (1,000 nm; 0.06%, 20 nm; 0.02%) for 9 h or with ATP (1 mM) for the last 3 h in stirred culture, and IL-1β and TNF-α production was then analyzed. Results are representative of 3 replicate experiments with triplicate cultures. Error bars represent s.d. within each group. (TIF) [file pone.0068499.s006.tif]

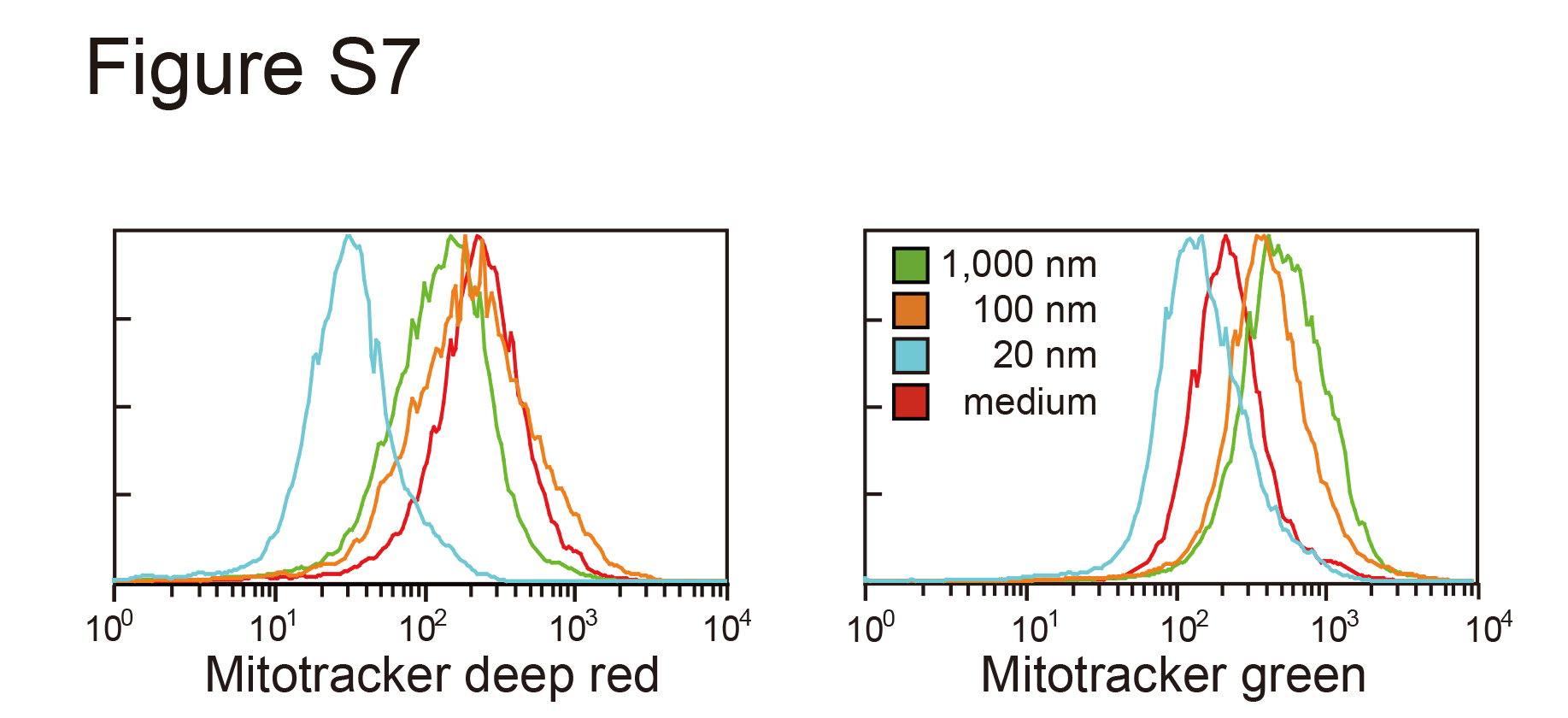

Supplement: Figure S7 — BMDM (BALB/c) were cultured with either LxBs (1,000 nm or 20 nm) or LPS alone, followed by the treatment with Mitotracker for 20 min as in Figure 4B, and the cells were then analyzed. (TIF) [file pone.0068499.s007.tif]

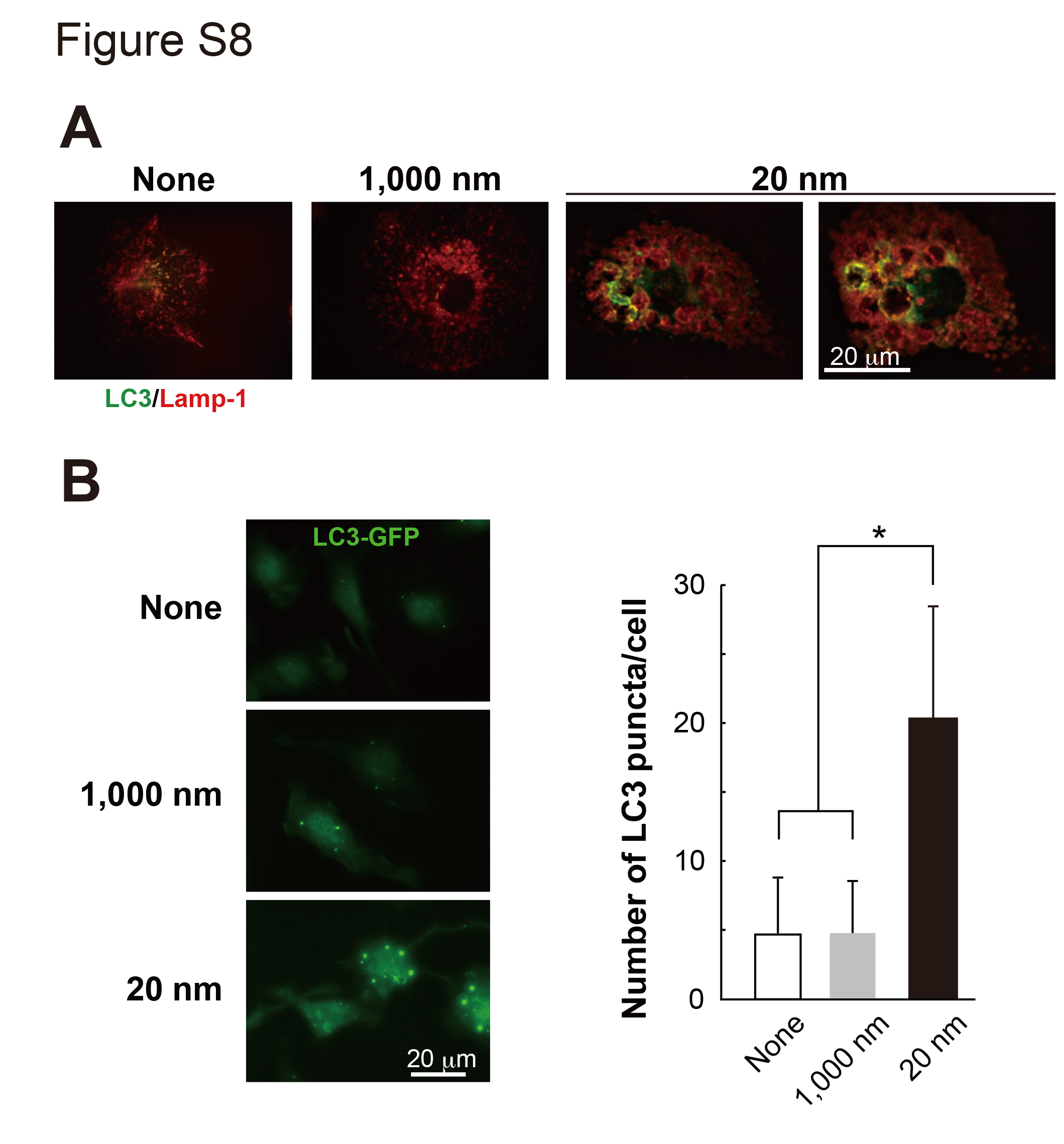

Supplement: Figure S8 — (A) As in Figure 1A, BMDM from LC3-GFP knock-in mice (C57BL/6) were cultured with 1,000 and 20 nm LxB (0.02%) for 18 h and stained with anti-Lamp-1 (red) and anti-GFP (green). For 20 nm LxB, two representative pictures of vacuolization are shown. (B) At 8 h after stimulation as in (A), BMDM were stained with anti-GFP (left panels), and the number of LC3-GFP+ puncta per cell was counted (right panel). *P < 0.05 (Tukey’s test). Error bars represent s.d. of 100 cells within each group. (TIF) [file pone.0068499.s008.tif]

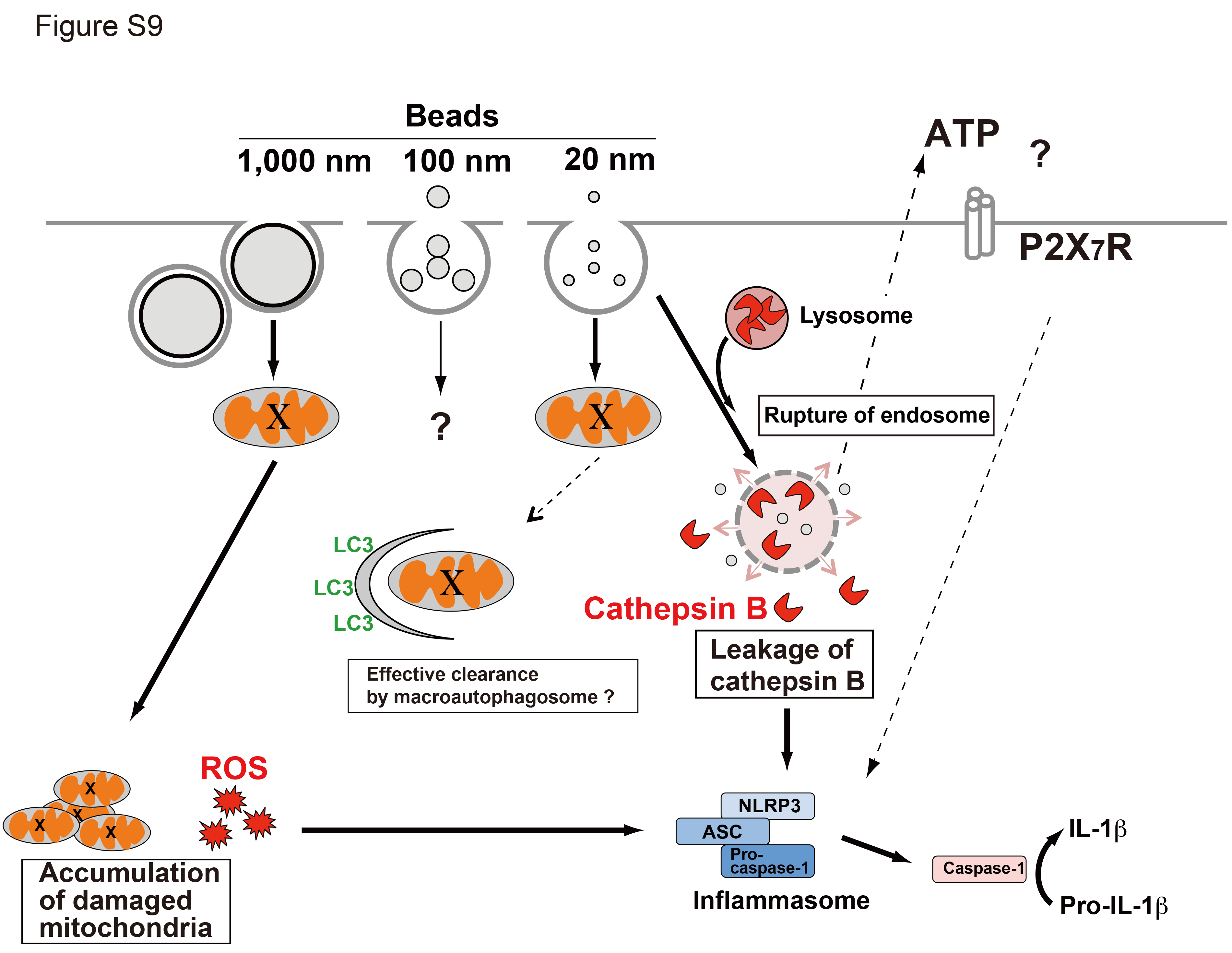

Supplement: Figure S9 — LxBs (1,000, 100 and 20 nm in diameter) that are endocytosed by macrophages cause mitochondria damage. Mitochondria damaged by 1.000 nm LxB seem to be cleared by a Parkin-dependent pathway, but not efficiently, leading to an accumulation of damaged mitochondria, production of ROS and activation of NLRP3 inflammasomes. On the other hand, 20 nm LxB cause rupture of the endosomes, leading to the release of cathepsin B (and possibly together with 20 nm LxB) into cytosol, followed by the activation of NLRP3 inflammasomes. In this case, damaged mitochondria may be effectively cleared by LC3+-macroautophagosomes or another unknown mechanism, resulting in no IL-1β production. LxB of 20 nm also cause release of ATP, possibly activating the P2X7-NLRP3 pathway. (TIF) [file pone.0068499.s009.tif]

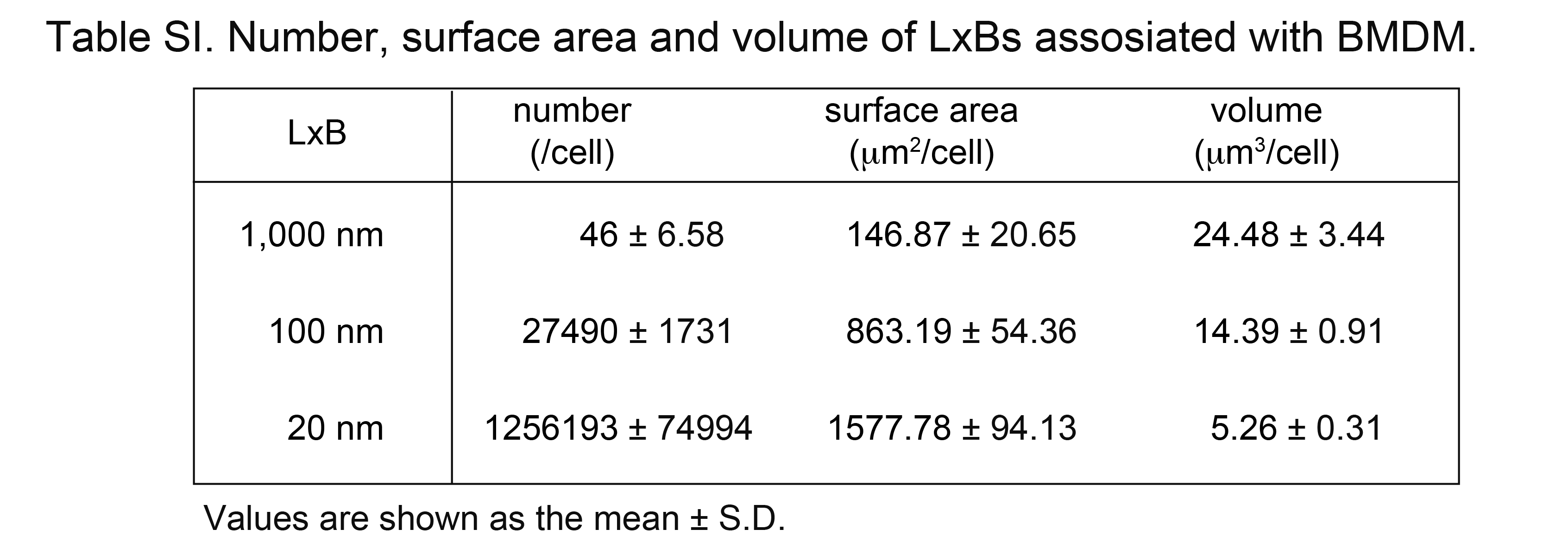

Supplement: Table S1 — (TIF) [file pone.0068499.s010.tif]
